# Supplementary material for: Functional classification of GNAI1 disorder variants in Caenorhabditis elegans uncovers conserved and cell-specific mechanisms of dysfunction
Source: Genetics. 2025 Oct 7;231(4):iyaf216. doi: 10.1093/genetics/iyaf216 (PMC12693566; doi:10.1093/genetics/iyaf216)
Supplement: iyaf216_Supplementary_Data [file iyaf216_supplementary_data.zip › Table_S1_GENETICS-2025-308494.docx]

**Table S1**: List of *C. elegans* strains used in this work

| **Strain** | **Genotype** | | **Source** |
| --- | --- | --- | --- |
| NWM868 | *odr-3(nch013[odr-3::wrmScarlet_11_])* V*; nchEx021[ceh-36△*p*::wrmScarlet_1-10_, unc-122△*p*::gfp]* | | This work |
| NWM447 | *oyls50[ceh-36*p*::gfp]* IV; *nchEx025[ceh-36△*p*::odr-3::tagrfp, unc-122△*p*::dsRed]* | | This work |
| NWM456 | *oyls50[ceh-36*p*::gfp]* IV; *nchEx026[ceh-36△*p*::odr-3^I321T^::tagrfp, unc-122△*p*::dsRed]* | | This work |
| NWM457 | *oyls50[ceh-36*p*::gfp]* IV; *nchEx027[ceh-36△*p*::odr-3^I321T^::tagrfp, unc-122△*p*::dsRed]* | | This work |
| NWM514 | *oyls50[ceh-36*p*::gfp]* IV; *nchEx033[ceh-36*△p*::odr-3^V334E^::tagrfp, unc-122△*p*::dsRed]* | | This work |
| NWM515 | *oyls50[ceh-36*p*::gfp]* IV; *nchEx034[ceh-36*△p*::odr-3^V334E^::tagrfp, unc-122△*p*::dsRed]* | | This work |
| NWM576 | *oyls50[ceh-36*p*::gfp]* IV; *nchEx019[ceh-36△*p*::odr-3^T48I^::tagrfp, unc-122△*p::*dsRed]* | | This work |
| NWM577 | *oyls50[ceh-36*p*::gfp]* IV; *nchEx020[ceh-36△*p*::odr-3^T48I^::tagrfp, unc-122△*p::*dsRed]* | | This work |
| NWM472 | *oyls50[ceh-36*p*::gfp]* IV; *nchEx022[ceh-36△*p*::odr-3^M88V^::tagrfp, unc-122△*p*::dsRed]* | | This work |
| NWM474 | *oyls50[ceh-36*p*::gfp]* IV; *nchEx023[ceh-36△*p*::odr-3^M88V^::tagrfp, unc-122△*p*::dsRed]* | | This work |
| NWM445 | *oyls50[ceh-36*p*::gfp]* IV; *nchEx035 [ceh-36△*p*::odr-3^D175V^::tagrfp, unc-122△*p*::dsRed]* | | This work |
| NWM446 | *oyls50[ceh-36*p*::gfp]* IV; *nchEx032 [ceh-36△*p*::odr-3^D175V^::tagrfp, unc-122△*p*::dsRed]* | | This work |
| NWM505 | *oyls50[ceh-36*p*::gfp]* IV; *nchEx030[ceh-36△*p*::odr-3^K272R^::tagrfp, unc-122△*p*::dsRed]* | | This work |
| NWM506 | *oyls50[ceh-36*p*::gfp]* IV; *nchEx031[ceh-36△*p*::odr-3^K272R^::tagrfp, unc-122△*p*::dsRed]* | | This work |
| NWM494 | *oyls50[ceh-36*p*::gfp]* IV; *nchEx028[ceh-36△*p*::odr-3^A328P^::tagrfp, unc-122△*p*::dsRed]* | | This work |
| NWM500 | *oyls50[ceh-36*p*::gfp]* IV; *nchEx029[ceh-36△*p*::odr-3^A328P^::tagrfp, unc-122△*p*::dsRed]* | | This work |
| NWM869 | *odr-3(nch027[odr-3[A328P]::wrmScarlet_11_])* V*; nchEx021[ceh-36△*p*::wrmScarlet_1-10_, unc-122△*p*::gfp]* | | This work |
| NWM862 | *mks-5(tm3100)* II*; oyls50[ceh-36*p*::gfp]* IV*; nchEx028[ceh-36△*p*::odr-3^A328P^::tagrfp, unc-122△*p*::dsRed]* | | This work |
| NWM861 | *mks-5(tm3100)* II*; oyls50[ceh-36*p*::gfp]* IV*; nchEx025[ceh-36△*p*::odr-3::tagrfp, unc-122△*p*::dsRed]* | | This work |
| PY3453 | *oyls50[ceh-36*p*::gfp]* IV | | (Kim, Kim et al. 2010) |
| NWM185 | *oyls50[ceh-36*p*::gfp]* IV; *odr-3(n1605)* V | | (Campagna, McMahon et al. 2023) |
| NWM448 | *oyls50[ceh-36*p*::gfp]* IV*; odr-3(nch004[M88V])* V | | This work |
| NWM502 | *oyls50[ceh-36*p*::gfp]* IV*; odr-3(nch005[I321T])* V | | This work |
| NWM563 | *oyls50[ceh-36*p*::gfp]* IV*; odr-3(nch012[T48I])* V | | This work |
| NWM545 | *oyls50[ceh-36*p*::gfp]* IV*; odr-3(nch011[D175V])* V | | This work |
| NWM544 | *oyls50[ceh-36*p*::gfp]* IV; *odr-3(nch009[A328P])* V | | This work |
| NWM540 | *oyls50[ceh-36*p*::gfp]* IV; *odr-3(nch0010[V334E])* V | | This work |
| NWM710 | *oyls50[ceh-36*p*::gfp]* IV; *odr-3 ^A328P^(nch009)* V*; nchEx025[ceh-36△*p*::odr-3::tagrfp, unc-122△*p*::dsRed]* | | This work |
| NWM652/653 | *oyls-50[ceh-36*p*::gfp]* IV; *odr-3(n1605)* V*; nchEx031[ceh-36△p::odr-3^K272R^::tagrfp, unc-122△*p*::dsRed]* | | This work |
| NWM864 | *nchEx042(bbs-8*p*::ric-8::vc155, ceh-36△*p*::vn173, unc-122△*p*::dsRed]* | | This work |
| NWM865 | *nchEx043(bbs-8*p*::ric-8::vc155, ceh-36△*p*::vn173, unc-122△*p*::dsRed]* | | This work |
| NWM765 | *nchEx038(bbs-8*p*::ric-8::vc155, ceh-36△*p*::odr-3::vn173, unc-122△*p*::dsRed]* | | This work |
| NWM767 | *nchEx039(bbs-8*p*::ric-8::vc155, ceh-36△*p*::odr-3::vn173, unc-122△*p*::dsRed]* | | This work |
| NWM831 | *nchEx040(bbs-8*p*::ric-8::vc155, ceh-36△*p*::odr-3^A328P^::vn173, unc-122△*p*::dsRed]* | | This work |
| NWM832 | *nchEx041(bbs-8*p*::ric-8::vc155, ceh-36△*p*::odr-3^A328P^::vn173, unc-122△*p*::dsRed]* | | This work |
| NWM858 | *nchEx045(ceh-36△*p*::ric-8::gfp, unc-122△*p*::dsRed)* | | This work |
| NWM859 | *nchEx046(ceh-36△*p*::ric-8::gfp, ceh-36△*p*::odr-3::tagrfp, unc-122△*p*::dsRed)* | | This work |
| NWM860 | *nchEx047(ceh-36△*p*::ric-8::gfp, ceh-36△*p*::odr-3^A328P^::tagrfp, unc-122△*p*::dsRed)* | | This work |
| NWM737 | *nchEx048(bbs-8*p*::vc155::unc-119, ceh-36△*p*::vn173, unc-122△*p*::dsRed]* | | This work |
| NWM738 | *nchEx049(bbs-8*p*::vc155::unc-119, ceh-36△*p*::vn173, unc-122△*p*::dsRed]* | | This work |
| NWM699 | *nchEx050(bbs-8*p*::vc155::unc-119, ceh-36△*p*::odr-3::vn173, unc-122△*p*::dsRed]* | | This work |
| NWM727 | *nchEx051(bbs-8*p*::vc155::unc-119, ceh-36△*p*::odr-3^A328P^::vn173, unc-122△*p*::dsRed]* | | This work |
| NWM728 | *nchEx052(bbs-8*p*::vc155::unc-119, ceh-36△*p*::odr-3^A328P^::vn173, unc-122△*p*::dsRed]* | | This work |
| NWM437 | *nchEx036[sra-6*p*::myr-gfp, sra-6*p*::mksr-2::tagrfp, unc-122△*p*::dsRed]* | | This work |
| NWM863 | *odr-3(n1605)* V*, nchEx036[sra-6△*p*::myr-gfp, sra-6△*p*::mksr-2::tagrfp, unc-122△*p*::dsRed]* | | This work |
| NWM709 | *nchEx037[sra-6*p*::myr-gfp, sra-6*p*::odr-3::tagrfp, unc-122△*p*::dsRed]* | | This work |
| NWM649 | *nchEx044[sra-6*p*::myr-gfp, sra-6*p*::odr-3^D175V^::tagrfp, unc-122△*p*::dsred]* | | This work |
| NWM711 | *odr-3(nch011[D175V])* V*; nchEx037[sra-6*p*::myr-gfp, sra-6*p*::odr-3::tagrfp, unc-122△*p*::dsRed]* | | This work |
|  |  |  | |
