# Supplementary material for: Functional classification of GNAI1 disorder variants in Caenorhabditis elegans uncovers conserved and cell-specific mechanisms of dysfunction
Source: Genetics. 2025 Oct 7;231(4):iyaf216. doi: 10.1093/genetics/iyaf216 (PMC12693566; doi:10.1093/genetics/iyaf216)
Supplement: iyaf216_Supplementary_Data [file iyaf216_supplementary_data.zip › Table_S2_GENETICS-2025-308494.docx]

| **Plasmid** | **Description** | **Source** |
| --- | --- | --- |
| NWM084 | *ceh-36Δ*p*::wrmScarlet_1-10_* | This work |
| Co-injection marker | *unc-122Δ*p*::gfp* | (Miyabayashi et al., 1999) |
| NWM032 | *ceh-36Δ*p*::odr-3∷tagrfp* | (Campagna et al., 2023) |
| Co-injection marker | *unc-122Δ*p*::dsRed* | (Miyabayashi et al., 1999) |
| NWM051 | *ceh-36Δ*p*::odr-3^I321T^::tagrfp* | This work |
| NWM055 | *ceh-36Δ*p*::odr-3^V334E^::tagrfp* | This work |
| NWM059 | *ceh-36Δ*p*::odr-3^T48I^::tagrfp* | This work |
| NWM050 | *ceh-36Δ*p*::odr-3^M88V^::tagrfp* | This work |
| NWM052 | *ceh-36Δ*p*::odr-3^D175V^::tagrfp* | This work |
| NWM053 | *ceh-36Δ*p*::odr-3^K272R^::tagrfp* | This work |
| NWM054 | *ceh-36Δ*p*::odr-3^A328P^::tagrfp* | This work |
| NWM036 | *bbs-8*p*::ric-8::vc155* | This work |
| NWM082 | *ceh-36Δ*p*::vn173* | (Campagna et al., 2023) |
| NWM034 | *ceh-36Δ*p*::odr-3^WT^::vn173* | (Campagna et al., 2023) |
| NWM095 | *ceh-36Δ*p*::odr-3^A328P^::vn173* | This work |
| NWM043 | *ceh-36Δ*p*::ric-8::gfp* | This work |
| NWM116 | *bbs-8*p*::vc155::unc-119* | This work |
| NWM100 | *sra-6p::myrgfp* | This work |
| NWM044 | *sra-6p::mksr-2::tagrfp* | This work |
| NWM097 | *sra-6*p*::odr-3^WT^::tagrfp* | This work |
| NWM099 | *sra-6*p*::odr-3^D175V^::tagrfp* | This work |
| NWM049 | *GNAI1^WT^::eGFP* *pcDNA3.1+* | This work |
| NWM065 | *GNAI1^D173V^::eGFP* *pcDNA3.1+* | This work |
| NWM066 | *GNAI1^K270R^::eGFP* *pcDNA3.1+* | This work |
| NWM068 | *GNAI1^A326P^::eGFP* *pcDNA3.1+* | This work |

**Table S2:** List of plasmids used in this work
