# Supplementary figures and images for: Functional classification of GNAI1 disorder variants in Caenorhabditis elegans uncovers conserved and cell-specific mechanisms of dysfunction
Source: Genetics. 2025 Oct 7;231(4):iyaf216. doi: 10.1093/genetics/iyaf216 (PMC12693566; doi:10.1093/genetics/iyaf216)

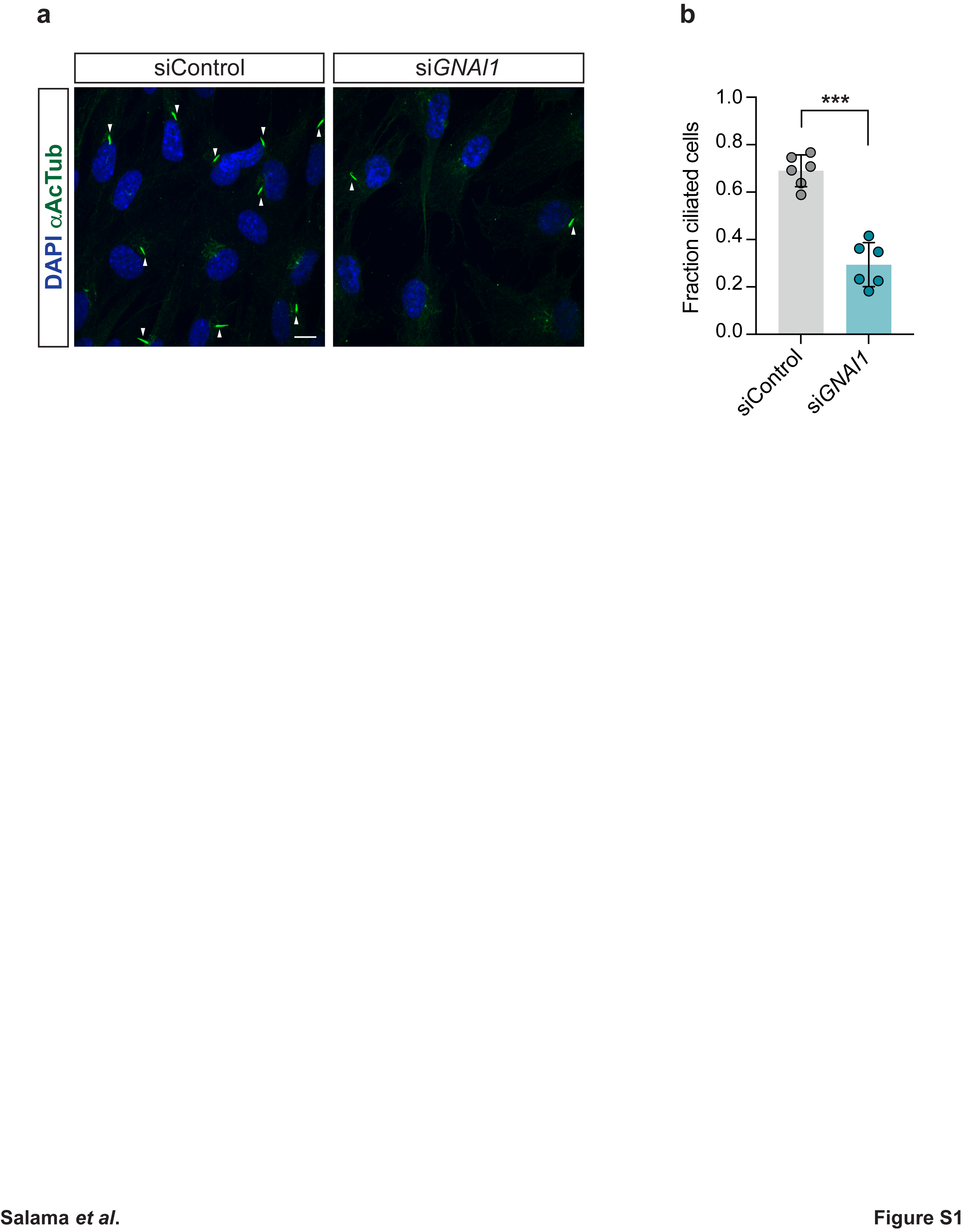

Supplement: iyaf216_Supplementary_Data [file iyaf216_supplementary_data.zip › Figure_S1_GENETICS-2025-308494.tif]

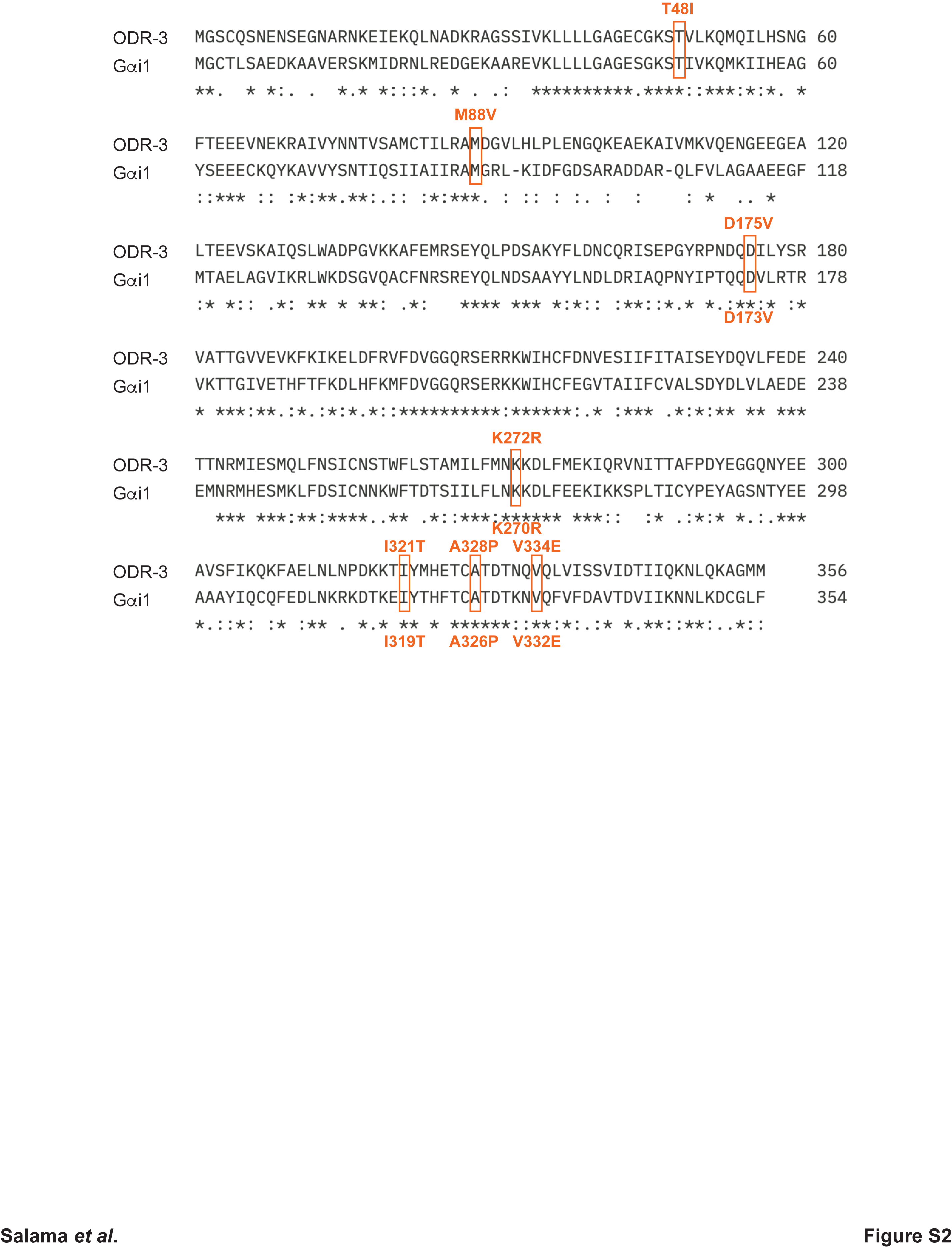

Supplement: iyaf216_Supplementary_Data [file iyaf216_supplementary_data.zip › Figure_S2_GENETICS-2025-308494.tif]

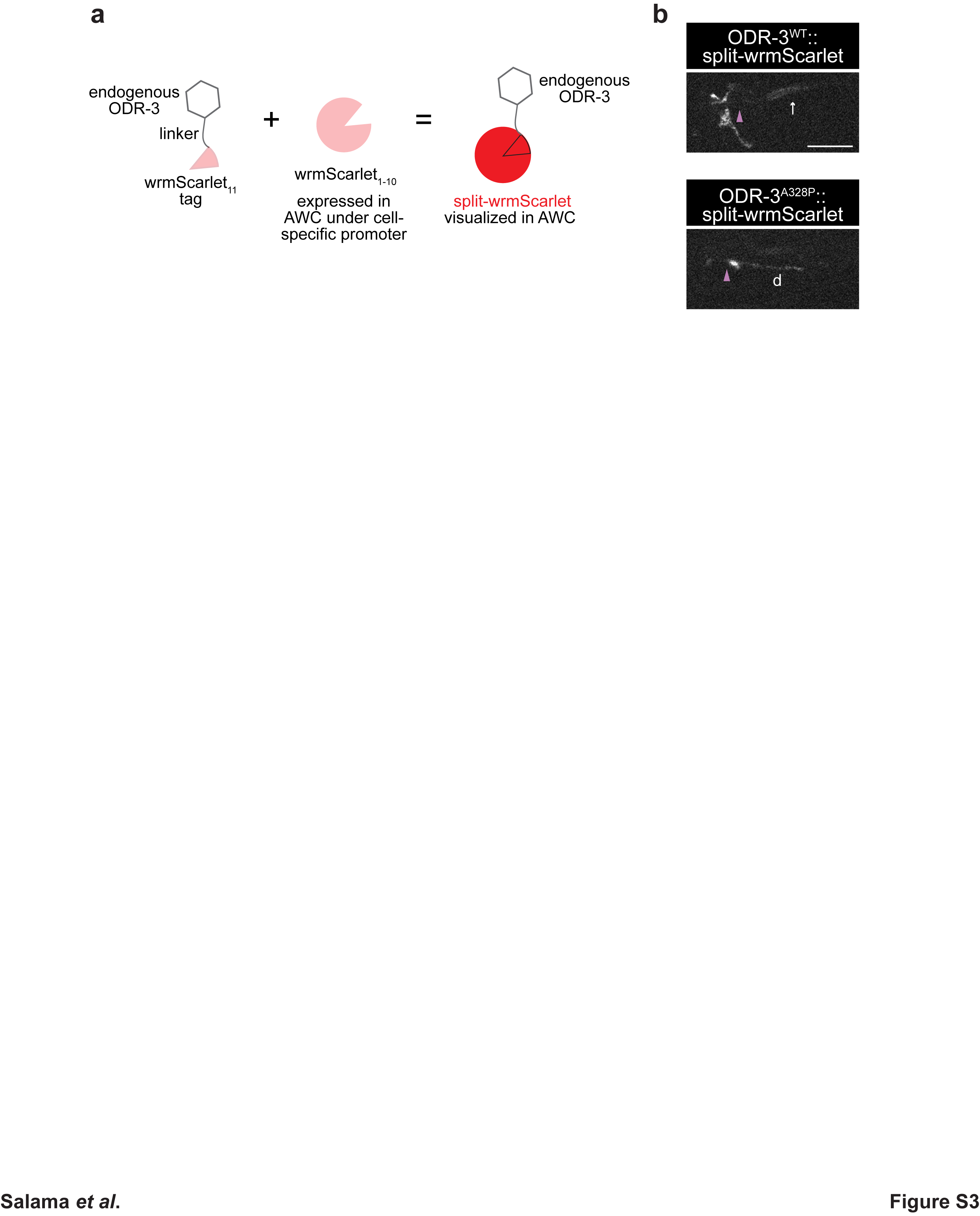

Supplement: iyaf216_Supplementary_Data [file iyaf216_supplementary_data.zip › Figure_S3_GENETICS-2025-308494.tif]

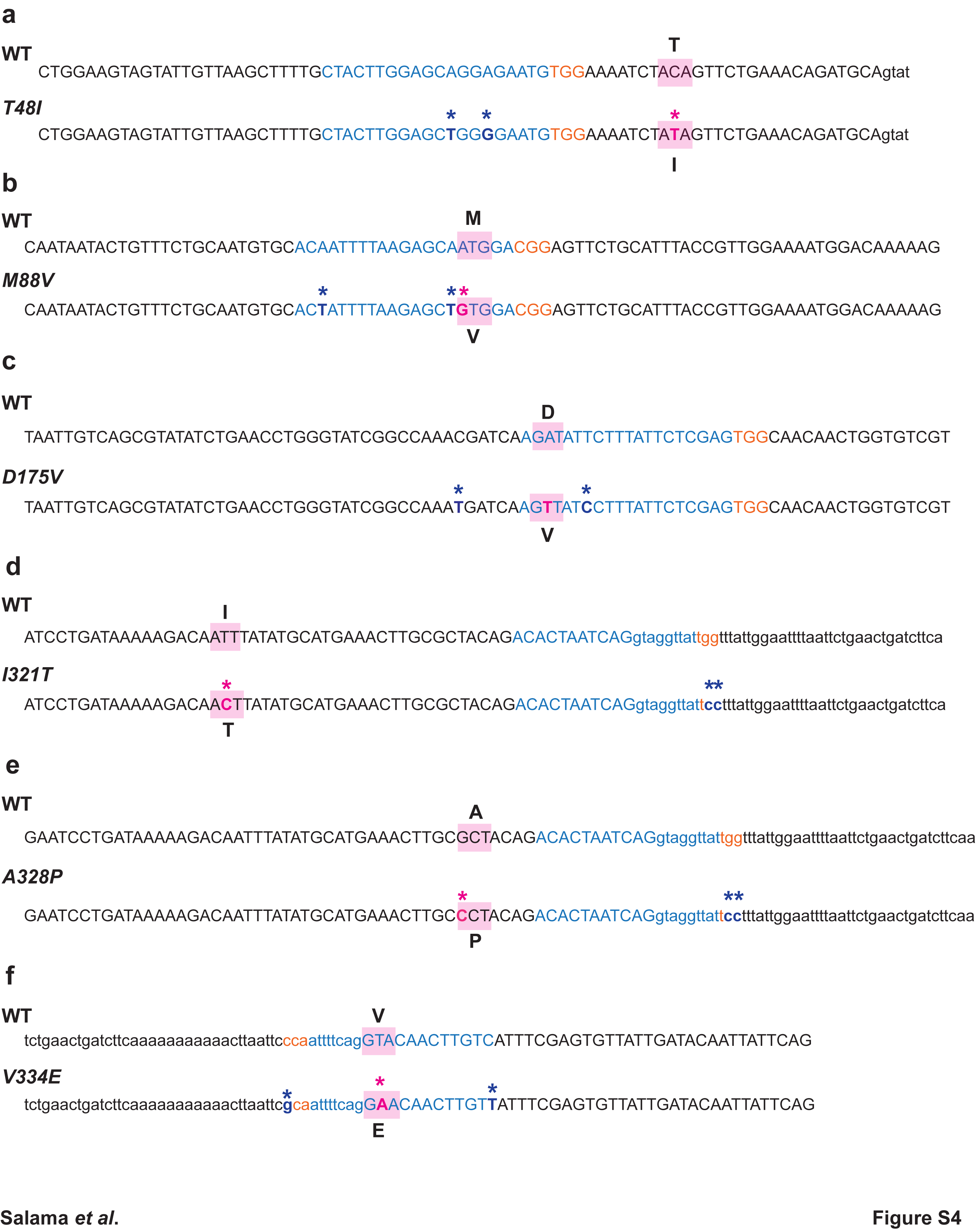

Supplement: iyaf216_Supplementary_Data [file iyaf216_supplementary_data.zip › Figure_S4_GENETICS-2025-308494.tif]

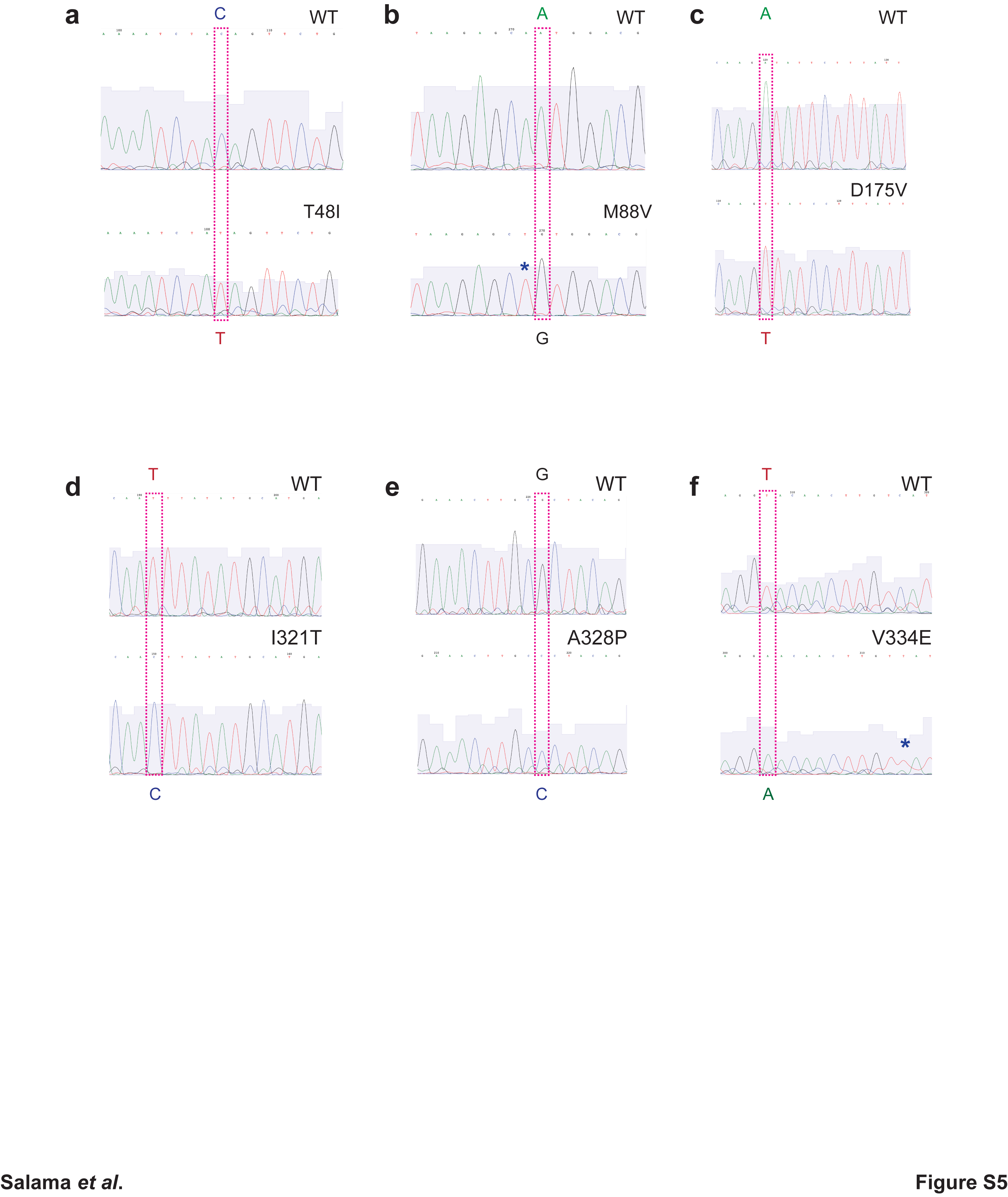

Supplement: iyaf216_Supplementary_Data [file iyaf216_supplementary_data.zip › Figure_S5_GENETICS-2025-308494.tif]

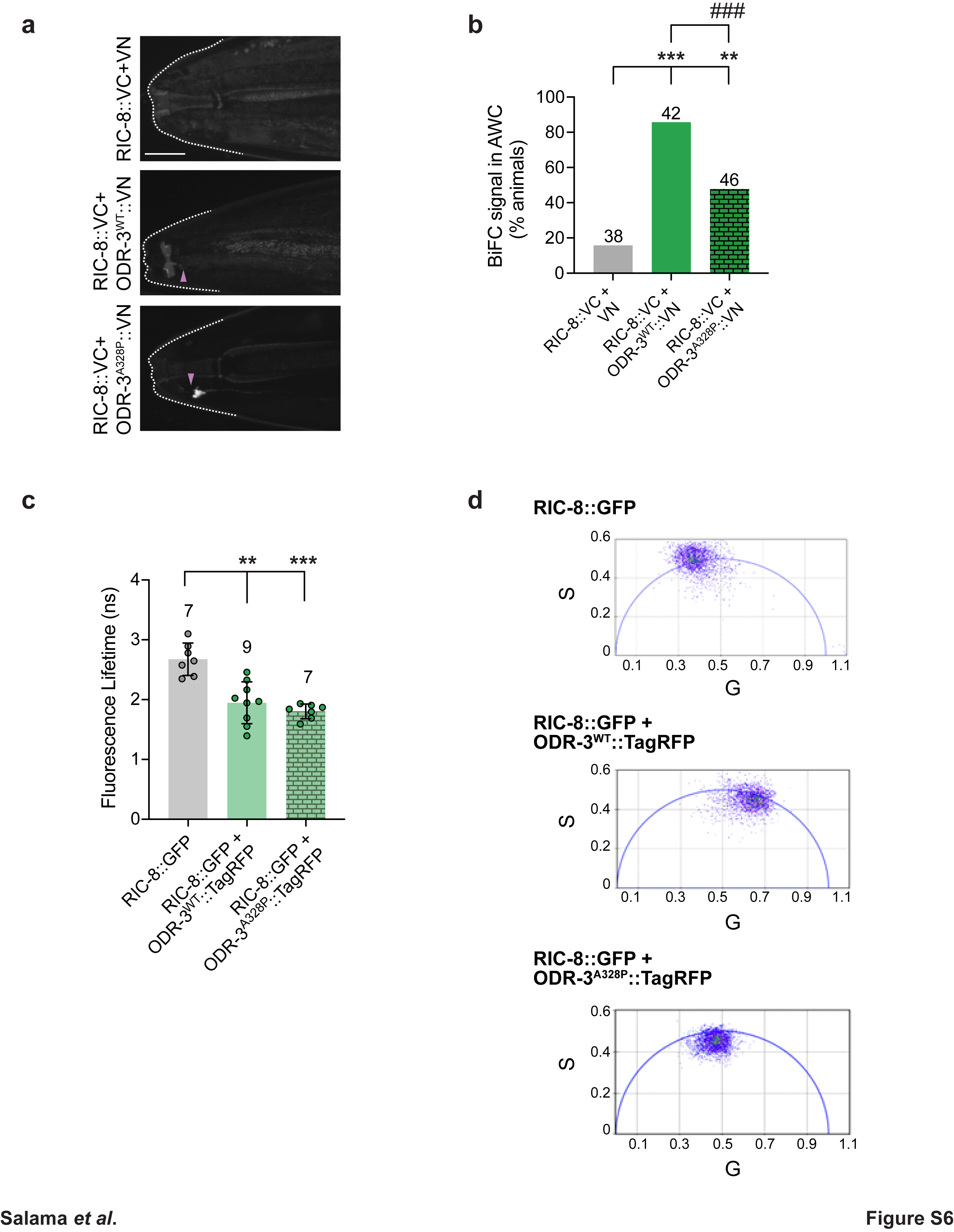

Supplement: iyaf216_Supplementary_Data [file iyaf216_supplementary_data.zip › Figure_S6_GENETICS-2025-308494.tif]

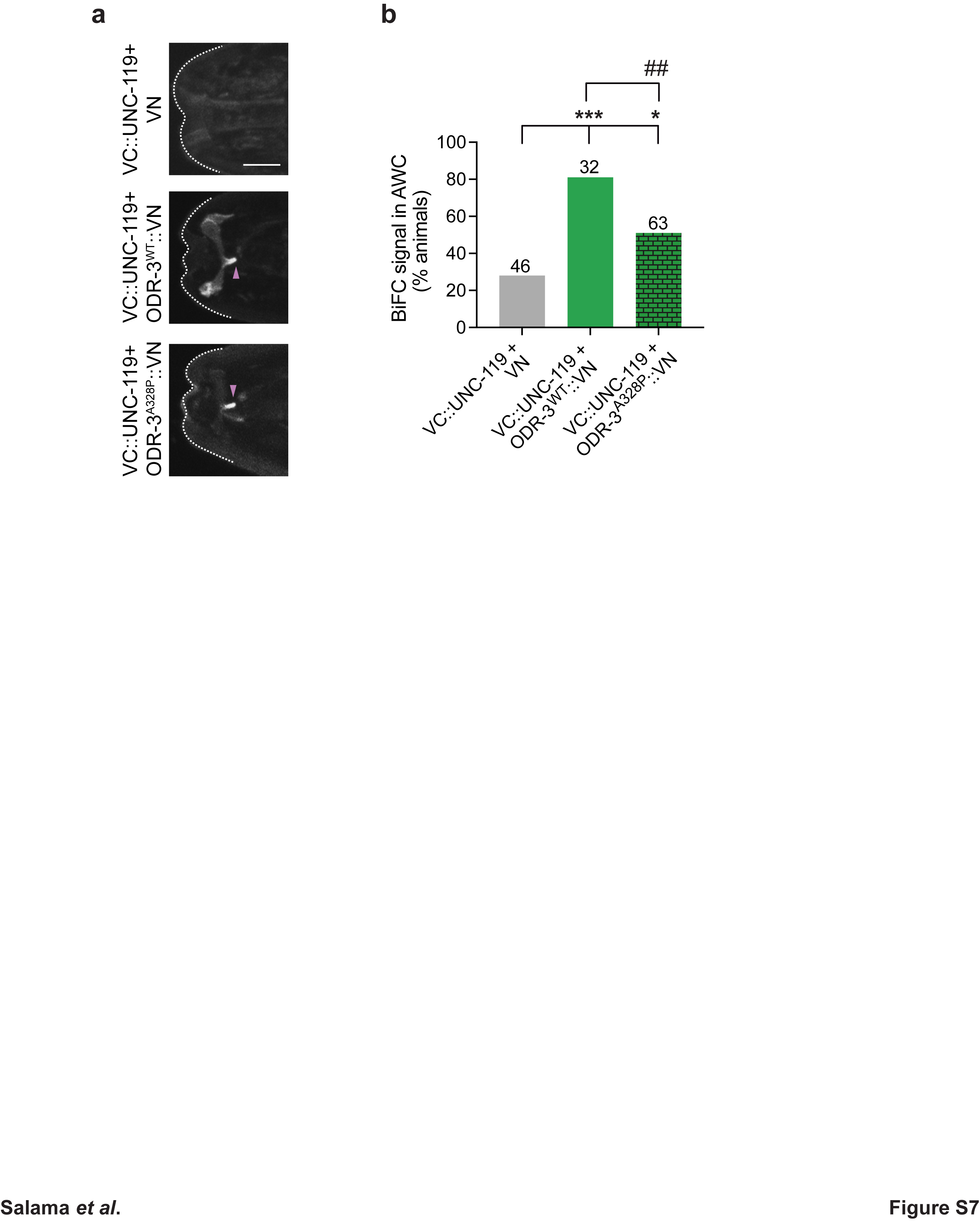

Supplement: iyaf216_Supplementary_Data [file iyaf216_supplementary_data.zip › Figure_S7_GENETICS-2025-308494.tif]

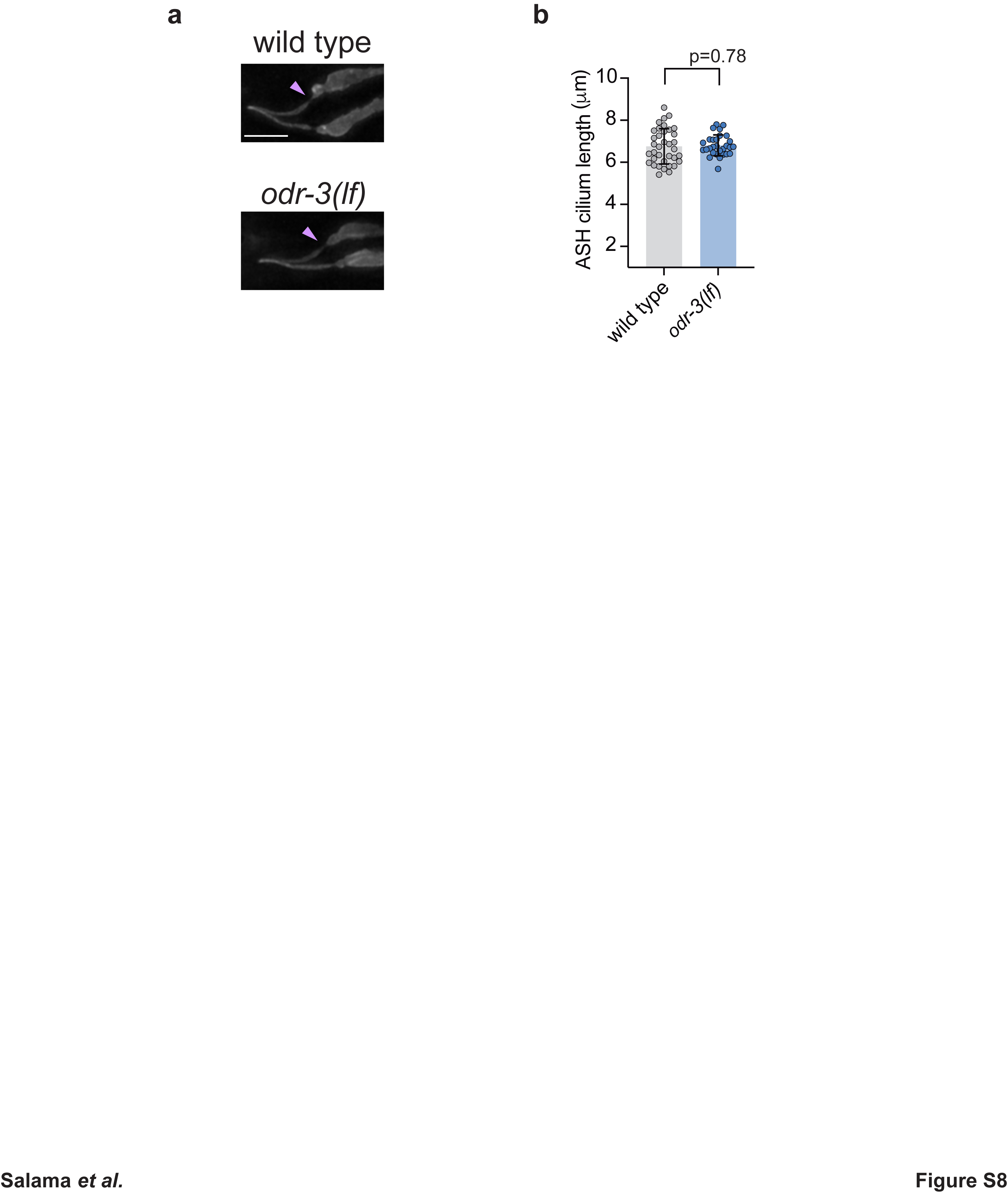

Supplement: iyaf216_Supplementary_Data [file iyaf216_supplementary_data.zip › Figure_S8_GENETICS-2025-308494.tif]
